# Supplementary material for: Atg1 kinase in fission yeast is activated by Atg11-mediated dimerization and cis-autophosphorylation
Source: eLife. 2020 Sep 10;9:e58073. doi: 10.7554/eLife.58073 (PMC7511232; doi:10.7554/eLife.58073)
Supplement: Supplementary file 1. [file elife-58073-supp1.docx]

**Supplementary File 1a. Fission yeast strains used in this study.**

| **Strains** | **Genotype** | |
| --- | --- | --- |
| LD328 | *h+* | *his3-D1 leu1-32* |
| DY47737 | *h+* | *leu1-32 Patg8-mYFP-atg8::kanMX* |
| DY47738 | *h?* | *leu1-32 atg1Δ::kanMX Patg8-mYFP-atg8::kanMX* |
| DY47739 | *h?* | *his3-D1 leu1-32 Patg8-mYFP-atg8::kanMX atg1-mCherry::kanMX* |
| DY47740 | *h?* | *his3-D1 leu1-32 Patg8-mYFP-atg8::kanMX atg1(D193A)-mCherry::kanMX* |
| DY47741 | *h?* | *his3-D1 leu1-32 Patg8-mYFP-atg8::kanMX atg1(T208A)-mCherry::kanMX* |
| DY47742 | *h+* | *his3-D1 leu1-32 atg1-mCherry::kanMX* |
| DY47743 | *h+* | *his3-D1 leu1-32 atg1(D193A)-mCherry::kanMX* |
| DY47744 | *h+* | *his3-D1 leu1-32 atg1(T208A)-mCherry::kanMX* |
| DY3953 | *h?* | *his3-D1 leu1-32 atg1-YFH::leu1+* |
| DY47745 | *h-* | *his3-D1 leu1-32 atg11Δ::kanMX atg1-YFH::leu1+* |
| DY3923 | *h?* | *his3-D1 leu1-32 atg13Δ::kanMX atg1-YFH::leu1+* |
| DY47746 | *h?* | *his3-D1 leu1-32 atg17Δ::kanMX atg1-YFH::leu1+* |
| DY47747 | *h?* | *his3-D1 leu1-32 atg101Δ::kanMX atg1-YFH::leu1+* |
| DY47748 | *h-* | *leu1-32::P41nmt1-mCherry-atg11(leu1+) atg11Δ::kanMX Patg8-mYFP-atg8::kanMX* |
| DY47749 | *h-* | *leu1-32::P41nmt1-mCherry-atg11(1-508)(leu1+) atg11Δ::kanMX Patg8-mYFP-atg8::kanMX* |
| DY47750 | *h-* | *leu1-32::P41nmt1-mCherry-atg11(508-926)(leu1+) atg11Δ::kanMX Patg8-mYFP-atg8::kanMX* |
| DY47751 | *h-* | *leu1-32::P41nmt1-mCherry-atg11(508-765)(leu1+) atg11Δ::kanMX Patg8-mYFP-atg8::kanMX* |
| DY47752 | *h-* | *leu1-32::Pnmt1-mCherry-atg11(522-583)(leu1+) atg11Δ::kanMX Patg8-mYFP-atg8::kanMX* |
| DY47753 | *h-* | *leu1-32::Pnmt1-mCherry-atg11(522-552)(leu1+) atg11Δ::kanMX Patg8-mYFP-atg8::kanMX* |
| DY47754 | *h-* | *leu1-32::Pnmt1-mCherry-atg11(532-583)(leu1+) atg11Δ::kanMX Patg8-mYFP-atg8::kanMX* |
| DY47755 | *h-* | *leu1-32::Pnmt1-mCherry-atg11(546-583)(leu1+) atg11Δ::kanMX Patg8-mYFP-atg8::kanMX* |
| DY47756 | *h-* | *leu1-32::P41nmt1-mCherry(leu1+) atg11Δ::kanMX Patg8-mYFP-atg8::kanMX* |
| DY47757 | *h-* | *leu1-32::P41nmt1-mCherry-atg11(522-583)Δ(leu1+) atg11Δ::kanMX Patg8-mYFP-atg8::kanMX* |
| DY47758 | *h?* | *ura4-D18 pho8Δ::hphMX atg11Δ::kanMX leu1-32::P41nmt1-pho8Δ60(S.cerevisiae)-GFP(leu1+) ars1::P41nmt1-mCherry(ura4+)* |
| DY47759 | *h?* | *ura4-D18 pho8Δ::hphMX atg11Δ::kanMX leu1-32::P41nmt1-pho8Δ60(S.cerevisiae)-GFP(leu1+) ars1::P41nmt1-mCherry-atg11(ura4+)* |
| DY47760 | *h?* | *ura4-D18 pho8Δ::hphMX atg11Δ::kanMX leu1-32::P41nmt1-pho8Δ60(S.cerevisiae)-GFP(leu1+) ars1::Pnmt1-mCherry-atg11(522-583)(ura4+)* |
| DY47761 | *h?* | *ura4-D18 pho8Δ::hphMX atg11Δ::kanMX leu1-32::P41nmt1-pho8Δ60(S.cerevisiae)-GFP(leu1+) ars1::P41nmt1-mCherry-atg11(522-583)Δ(ura4+)* |
| DY47762 | *h-* | *leu1-32 his3-D1::P41nmt1-mCherry(his+) atg11Δ::kanMX atg1-YFH::leu1+* |
| DY47763 | *h?* | *his3-D1 leu1-32::P41nmt1-mCherry-atg11(leu1+) atg11Δ::kanMX atg1-YFH::leu1+* |
| DY47764 | *h?* | *his3-D1 leu1-32 ars1::Pnmt1-mCherry-atg11(522-583)(ura4+) atg11Δ::kanMX atg1-YFH::leu1+* |
| DY47765 | *h?* | *his3-D1 leu1-32 ars1::P41nmt1-mCherry-atg11(522-583)Δ(ura4+) atg11Δ::kanMX atg1-YFH::leu1+* |
| DY3959 | *h?* | *his3-D1 leu1-32 atg11-YFH::leu1+* |
| DY47766 | *h-* | *his3-D1 leu1-32 atg1-myc::hphMX* |
| DY47767 | *h+* | *his3-D1 leu1-32 atg1-myc::hphMX atg11-YFH::leu1+* |
| DY47768 | *h+* | *his3-D1 ura4-D18 leu1-32::P41nmt1-Pil1-CFP-atg11(522-583)(leu+) ars1::P41nmt1-mCherry-atg1(1-311)(ura4+)* |
| DY47769 | *h+* | *his3-D1 ura4-D18 leu1-32::P41nmt1-Pil1-CFP-atg11(522-583)(leu+) ars1::P41nmt1-mCherry-atg1(312-592)(ura4+)* |
| DY47770 | *h+* | *his3-D1 ura4-D18 leu1-32::P41nmt1-Pil1-CFP-atg11(522-583)(leu+) ars1::P41nmt1-mCherry-atg1(593-830)(ura4+)* |
| DY47771 | *h+* | *his3-D1 ura4-D18 leu1-32::P41nmt1-Pil1-CFP-atg11(522-552)(leu+) ars1::P41nmt1-mCherry-atg1(593-830)(ura4+)* |
| DY47772 | *h+* | *his3-D1 ura4-D18 leu1-32::P41nmt1-Pil1-CFP-atg11(532-583)(leu+) ars1::P41nmt1-mCherry-atg1(593-830)(ura4+)* |
| DY47773 | *h-* | *leu1-32::Pnmt1-mCherry-atg11(522-583)F526A(leu1+) atg11Δ::kanMX Patg8-mYFP-atg8::kanMX* |
| DY47774 | *h-* | *leu1-32::Pnmt1-mCherry-atg11(522-583)Y527A(leu1+) atg11Δ::kanMX Patg8-mYFP-atg8::kanMX* |
| DY47775 | *h-* | *leu1-32::Pnmt1-mCherry-atg11(522-583)F526A Y527A(leu1+) atg11Δ::kanMX Patg8-mYFP-atg8::kanMX* |
| DY47776 | *h?* | *his3-D1 ura4-D18 isp6Δ::hphMX psp3Δ::kanMX leu1-32::Pnmt1-mCherry-atg11(522-583)(leu1+)* |
| DY47777 | *h?* | *his3-D1 ura4-D18 leu1-32 isp6Δ::hphMX psp3Δ::kanMX ars1::Pnmt1-atg11(522-583)-GFP(ura4+)* |
| DY47778 | *h?* | *his3-D1 ura4-D18 isp6Δ::hphMX psp3Δ::kanMX leu1-32::Pnmt1-mCherry-atg11(522-583)(leu1+) ars1::Pnmt1-atg11(522-583)-GFP(ura4+)* |
| DY47779 | *h-* | *ura4-D18 leu1-32::Pnmt1-mCherry-atg11(522-552)(leu1+)* |
| DY47780 | *h+* | *ura4-D18 leu1-32 ars1::Pnmt1-atg11(522-552)-GFP(ura4+)* |
| DY47781 | *h?* | *ura4-D18 leu1-32::Pnmt1-mCherry-atg11(522-552)(leu1+) ars1::Pnmt1-atg11(522-552)-GFP(ura4+)* |
| DY47782 | *h?* | *his3-D1 ura4-D18 isp6Δ::hphMX psp3Δ::kanMX leu1-32::Pnmt1-mCherry-atg11(546-583)(leu1+)* |
| DY47783 | *h?* | *his3-D1 ura4-D18 leu1-32 isp6Δ::hphMX psp3Δ::kanMX ars1::Pnmt1-atg11(546-583)-GFP(ura4+)* |
| DY47784 | *h?* | *his3-D1 ura4-D18 isp6Δ::hphMX psp3Δ::kanMX leu1-32::Pnmt1-mCherry-atg11(546-583)(leu1+) ars1::Pnmt1-atg11(546-583)-GFP(ura4+)* |
| DY47785 | *h-* | *leu1-32::P41nmt1-mCherry-atg11(522-583)(leu1+) atg11Δ::kanMX Patg8-mYFP-atg8::kanMX* |
| DY47786 | *h-* | *leu1-32::P81nmt1-mCherry-atg11(522-583)-Leucine_zipper(leu1+) atg11Δ::kanMX Patg8-mYFP-atg8::kanMX* |
| DY47787 | *h-* | *leu1-32::P81nmt1-mCherry-atg11(522-552)-Leucine_zipper(leu1+) atg11Δ::kanMX Patg8-mYFP-atg8::kanMX* |
| DY47788 | *h-* | *leu1-32::P41nmt1-mCherry-atg11(532-583)-Leucine_zipper(leu1+) atg11Δ::kanMX Patg8-mYFP-atg8::kanMX* |
| DY47789 | *h?* | *ura4-D18 pho8Δ::hphMX atg11Δ::kanMX leu1-32::P41nmt1-pho8Δ60(S.cerevisiae)-GFP(leu1+) ars1::P41nmt1-mCherry-atg11(522-583)(ura4+)* |
| DY47790 | *h?* | *ura4-D18 pho8Δ::hphMX atg11Δ::kanMX leu1-32::P41nmt1-pho8Δ60(S.cerevisiae)-GFP(leu1+) ars1::Pnmt1-mCherry-atg11(522-552)(ura4+)* |
| DY47791 | *h?* | *ura4-D18 pho8Δ::hphMX atg11Δ::kanMX leu1-32::P41nmt1-pho8Δ60(S.cerevisiae)-GFP(leu1+) ars1::Pnmt1-mCherry-atg11(532-583)(ura4+)* |
| DY47792 | *h?* | *ura4-D18 pho8Δ::hphMX atg11Δ::kanMX leu1-32::P41nmt1-pho8Δ60(S.cerevisiae)-GFP(leu1+) ars1::P81nmt1-mCherry-atg11(522-583)-Leucine_zipper(ura4+)* |
| DY47793 | *h?* | *ura4-D18 pho8Δ::hphMX atg11Δ::kanMX leu1-32::P41nmt1-pho8Δ60(S.cerevisiae)-GFP(leu1+) ars1::P81nmt1-mCherry-atg11(522-552)-Leucine_zipper(ura4+)* |
| DY47794 | *h?* | *ura4-D18 pho8Δ::hphMX atg11Δ::kanMX leu1-32::P41nmt1-pho8Δ60(S.cerevisiae)-GFP(leu1+) ars1::P41nmt1-mCherry-atg11(532-583)-Leucine_zipper(ura4+)* |
| DY47795 | *h?* | *leu1-32 atg1Δ::kanMX atg11Δ::hphMX Patg8-mYFP-atg8::kanMX* |
| DY47796 | *h?* | *leu1-32::Patg1-mCherry-atg1(leu1+) atg1Δ::kanMX atg11Δ::hphMX Patg8-mYFP-atg8::kanMX* |
| DY47797 | *h?* | *leu1-32::Patg1-mCherry-atg1-Leucine_zipper(leu1+) atg1Δ::kanMX atg11Δ::hphMX Patg8-mYFP-atg8::kanMX* |
| DY47798 | *h?* | *his3-D1 ura4-D18 atg1Δ::natMX atg11Δ::kanMX pho8Δ::bsdMX pha2::P41nmt1-pho8Δ60(S.cerevisiae)(hphMX)(pha2-) leu1-32::Patg1-GBP(leu1+) ars1::Patg1-atg1-GFP(ura4+)* |
| DY47799 | *h?* | *his3-D1 ura4-D18 atg1Δ::natMX atg11Δ::kanMX pho8Δ::bsdMX pha2::P41nmt1-pho8Δ60(S.cerevisiae)(hphMX)(pha2-) leu1-32::Patg1-atg1-GBP(leu1+) ars1::Patg1-atg1-GFP(ura4+)* |
| DY47800 | *h?* | *his3-D1 ura4-D18 atg1Δ::natMX atg11Δ::kanMX pho8Δ::bsdMX pha2::P41nmt1-pho8Δ60(S.cerevisiae)(hphMX) leu1-32::Patg1-atg1(D193A)-GBP(leu1+) ars1::Patg1-atg1-GFP(ura4+)* |
| DY47801 | *h?* | *his3-D1 ura4-D18 atg1Δ::natMX atg11Δ::kanMX pho8Δ::bsdMX pha2::P41nmt1-pho8Δ60(S.cerevisiae)(hphMX) leu1-32::Patg1-atg1(T208A)-GBP(leu1+) ars1::Patg1-atg1-GFP(ura4+)* |
| DY47802 | *h?* | *his3-D1 ura4-D18 atg1Δ::natMX atg11Δ::kanMX pho8Δ::bsdMX pha2::P41nmt1-pho8Δ60(S.cerevisiae)(hphMX) leu1-32::Patg1-atg1(D193AT208A)-GBP(leu1+) ars1::Patg1-atg1-GFP(ura4+)* |
| DY47803 | *h?* | *his3-D1 ura4-D18 atg1Δ::natMX atg11Δ::kanMX pho8Δ::bsdMX pha2::P41nmt1-pho8Δ60(S.cerevisiae)(hphMX) leu1-32::Patg1-atg1-Flag-GBP(leu1+) ars1::Patg1-atg1(D193A)-GFP(ura4+)* |
| DY47804 | *h?* | *his3-D1 ura4-D18 atg1Δ::natMX atg11Δ::kanMX pho8Δ::bsdMX pha2::Ppha2-nmt41-pho8Δ60(S.cerevisiae)(hphMX) leu1-32::Patg1-atg1(D193A)-Flag-GBP(leu1+) ars1::Patg1-atg1(D193A)-GFP(ura4+)* |
| DY47805 | *h?* | *his3-D1 ura4-D18 atg1Δ::natMX atg11Δ::kanMX pho8Δ::bsdMX pha2::P41nmt1-pho8Δ60(S.cerevisiae)(hphMX) leu1-32::Patg1-atg1(T208A)-GBP(leu1+) ars1::Patg1-atg1(D193A)-GFP(ura4+)* |
| DY47806 | *h?* | *his3-D1 ura4-D18 atg1Δ::natMX atg11Δ::kanMX pho8Δ::bsdMX pha2::P41nmt1-pho8Δ60(S.cerevisiae)(hphMX) leu1-32::Patg1-atg1-Flag-GBP(leu1+) ars1::Patg1-atg1(T208A)-GFP(ura4+)* |
| DY47807 | *h?* | *his3-D1 ura4-D18 atg1Δ::natMX atg11Δ::kanMX pho8Δ::bsdMX pha2::P41nmt1-pho8Δ60(S.cerevisiae)(hphMX) leu1-32::Patg1-atg1(D193A)-Flag-GBP(leu1+) ars1::Patg1-atg1(T208A)-GFP(ura4+)* |
| DY47808 | *h?* | *his3-D1 ura4-D18 atg1Δ::natMX atg11Δ::kanMX pho8Δ::bsdMX pha2::P41nmt1-pho8Δ60(S.cerevisiae)(hphMX) leu1-32::Patg1-atg1(T208A)-GBP(leu1+) ars1::Patg1-atg1(T208A)-GFP(ura4+)* |
| DY47809 | *h+* | *his3-D1 leu1-32::Pnmt41-mCherry-atg11(leu1+)* |
| DY47810 | *h?* | *his3-D1 leu1-32::Pnmt41-mCherry-atg11(leu1+) atg11-YFH::leu1+* |
| DY47811 | *h?* | *his3-D1 leu1-32::Pnmt41-mCherry-atg11(leu1+) atg1Δ::kanMX atg11-YFH::leu1+* |
| DY47812 | *h+* | *leu1-32 ura4-D18 ars1::Pnmt1-atg11(546-583)Δ-GFP(ura4+)* |
| DY47813 | *h+* | *ura4-D18 leu1-32::Pnmt1-mCherry-atg11(546-583)Δ(leu1+)* |
| DY47814 | *h+* | *ura4-D18 leu1-32::Pnmt1-mCherry-atg11(546-583)Δ(leu1+) ars1::Pnmt1-atg11(546-583)Δ(ura4+)* |
| DY47815 | *h-* | *leu1-32::Pnmt41-mCherry-atg11(546-583)Δ(leu1+) atg11Δ::kanMX Patg8-mYFP-atg8::kanMX* |
| DY47816 | *h?* | *his3-D1 leu1-32::Pnmt41-mCherry-atg11(546-583)Δ(leu1+) atg11Δ::kanMX atg1-YFH::leu1+* |
| DY47817 | *h?* | *his3-D1 ura4-D18 atg11Δ::kanMX leu1-32::Pnmt1-Erg11-CFP(leu1+) ars1::Pnmt41-mCherry-atg11(ura4+)* |
| DY47818 | *h?* | *his3-D1 ura4-D18 atg11Δ::kanMX leu1-32::Pnmt1-Erg11-CFP(leu1+) ars1::Pnmt41-mCherry-atg11(522-583)(ura4+)* |
| DY47819 | *h?* | *his3-D1 ura4-D18 atg11Δ::kanMX leu1-32::Pnmt1-Erg11-CFP(leu1+) ars1::Pnmt1-mCherry-atg11(522-552)(ura4+)* |
| DY47820 | *h?* | *his3-D1 ura4-D18 atg11Δ::kanMX leu1-32::Pnmt1-Erg11-CFP(leu1+) ars1::Pnmt81-mCherry-atg11(522-552)-LZ(ura4+)* |
| DY47821 | *h?* | *his3-D1 ura4-D18 atg11Δ::kanMX leu1-32::Pnmt1-Erg11-CFP(leu1+) ars1::Pnmt41-mCherry(ura4+)* |
| DY47822 | *h?* | *his3-D1 leu1-32::Pnmt1-Erg11-CFP(leu1+)* |
| DY47446 | *h?* | *his3-D1 epr1Δ::kanMX leu1-32::Pnmt1-Erg11-CFP(leu1+) Cpy1-mCherry::natMX* |
| DY47823 | *h+* | *his3-D1 ura4-D18 atg1Δ::natMX pha2::P41nmt1-mCherry-atg8(hphMX)(pha2-) leu1-32::Patg1-GBP(leu1+)* |
| DY47824 | *h+* | *his3-D1 ura4-D18 atg1Δ::natMX pha2::P41nmt1-mCherry-atg8(hphMX)(pha2-) leu1-32::Patg1-atg1-GBP(leu1+)* |
| DY47825 | *h+* | *his3-D1 ura4-D18 atg1Δ::natMX pha2::P41nmt1-mCherry-atg8(hphMX)(pha2-) leu1-32::Patg1-atg1(D193A)-GBP(leu1+)* |
| DY47826 | *h+* | *his3-D1 ura4-D18 atg1Δ::natMX pha2::P41nmt1-mCherry-atg8(hphMX)(pha2-) leu1-32::Patg1-atg1(T208A)-GBP(leu1+)* |
| DY47827 | *h+* | *his3-D1 ura4-D18 atg1Δ::natMX pha2::P41nmt1-mCherry-atg8(hphMX)(pha2-) leu1-32::Patg1-atg1(D193A T208A)-GBP(leu1+)* |
| DY47828 | *h+* | *his3-D1 ura4-D18 atg1Δ::natMX pha2::P41nmt1-mCherry-atg8(hphMX)(pha2-) leu1-32::Patg1-GFP(leu1+)* |
| DY47829 | *h+* | *his3-D1 ura4-D18 atg1Δ::natMX pha2::P41nmt1-mCherry-atg8(hphMX)(pha2-) leu1-32::Patg1-atg1-GFP(leu1+)* |
| DY47830 | *h+* | *his3-D1 ura4-D18 atg1Δ::natMX pha2::P41nmt1-mCherry-atg8(hphMX)(pha2-) leu1-32::Patg1-atg1(D193A)-GFP(leu1+)* |
| DY47831 | *h+* | *his3-D1 ura4-D18 atg1Δ::natMX pha2::P41nmt1-mCherry-atg8(hphMX)(pha2-) leu1-32::Patg1-atg1(T208A)-GFP(leu1+)* |
| DY47832 | *h+* | *his3-D1 ura4-D18 atg1Δ::natMX pha2::P41nmt1-mCherry-atg8(hphMX)(pha2-) leu1-32::Patg1-atg1(D193A T208A)-GFP(leu1+)* |
| DY47833 | *h-* | *his3-D1 ura4-D18 atg1Δ::natMX atg11Δ::kanMX pha2::P41nmt1-mCherry-atg8(hphMX)(pha2-) leu1-32::Patg1-GBP(leu1+)* |
| DY47834 | *h-* | *his3-D1 ura4-D18 atg1Δ::natMX atg11Δ::kanMX pha2::P41nmt1-mCherry-atg8(hphMX)(pha2-) leu1-32::Patg1-atg1-GBP(leu1+)* |
| DY47835 | *h-* | *his3-D1 ura4-D18 atg1Δ::natMX atg11Δ::kanMX pha2::P41nmt1-mCherry-atg8(hphMX)(pha2-) leu1-32::Patg1-atg1(D193A)-GBP(leu1+)* |
| DY47836 | *h-* | *his3-D1 ura4-D18 atg1Δ::natMX atg11Δ::kanMX pha2::P41nmt1-mCherry-atg8(hphMX)(pha2-) leu1-32::Patg1-atg1(T208A)-GBP(leu1+)* |
| DY47837 | *h-* | *his3-D1 ura4-D18 atg1Δ::natMX atg11Δ::kanMX pha2::P41nmt1-mCherry-atg8(hphMX)(pha2-) leu1-32::Patg1-atg1(D193A T208A)-GBP(leu1+)* |
| DY47838 | *h-* | *his3-D1 ura4-D18 atg1Δ::natMX atg11Δ::kanMX pha2::P41nmt1-mCherry-atg8(hphMX)(pha2-) leu1-32::Patg1-GFP(leu1+)* |
| DY47839 | *h-* | *his3-D1 ura4-D18 atg1Δ::natMX atg11Δ::kanMX pha2::P41nmt1-mCherry-atg8(hphMX)(pha2-) leu1-32::Patg1-atg1-GFP(leu1+)* |
| DY47840 | *h-* | *his3-D1 ura4-D18 atg1Δ::natMX atg11Δ::kanMX pha2::P41nmt1-mCherry-atg8(hphMX)(pha2-) leu1-32::Patg1-atg1(D193A)-GFP(leu1+)* |
| DY47841 | *h-* | *his3-D1 ura4-D18 atg1Δ::natMX atg11Δ::kanMX pha2::P41nmt1-mCherry-atg8(hphMX)(pha2-) leu1-32::Patg1-atg1(T208A)-GFP(leu1+)* |
| DY47842 | *h-* | *his3-D1 ura4-D18 atg1Δ::natMX atg11Δ::kanMX pha2::P41nmt1-mCherry-atg8(hphMX)(pha2-) leu1-32::Patg1-atg1(D193A T208A)-GFP(leu1+)* |
| DY47843 | *h-* | *his3-D1 ura4-D18 atg1Δ::natMX atg11Δ::kanMX pha2::P41nmt1-mCherry-atg8(hphMX)(pha2-) leu1-32::Patg1-GBP(leu1+) ars1::Patg1-atg1-GFP(ura4+)* |
| DY47844 | *h-* | *his3-D1 ura4-D18 atg1Δ::natMX atg11Δ::kanMX pha2::P41nmt1-mCherry-atg8(hphMX)(pha2-) leu1-32::Patg1-atg1-GBP(leu1+) ars1::Patg1-atg1-GFP(ura4+)* |
| DY47845 | *h-* | *his3-D1 ura4-D18 atg1Δ::natMX atg11Δ::kanMX pha2::P414nmt1-mCherry-atg8(hphMX)(pha2-) leu1-32::Patg1-atg1(D193A)-GBP(leu1+) ars1::Patg1-atg1-GFP(ura4+)* |
| DY47846 | *h-* | *his3-D1 ura4-D18 atg1Δ::natMX atg11Δ::kanMX pha2::P41nmt1-mCherry-atg8(hphMX)(pha2-) leu1-32::Patg1-atg1(T208A)-GBP(leu1+) ars1::Patg1-atg1-GFP(ura4+)* |
| DY47847 | *h-* | *his3-D1 ura4-D18 atg1Δ::natMX atg11Δ::kanMX pha2::P41nmt1-mCherry-atg8(hphMX)(pha2-) leu1-32::Patg1-atg1(D193AT208A)-GBP(leu1+) ars1::Patg1-atg1-GFP(ura4+)* |
| DY47848 | *h-* | *his3-D1 ura4-D18 atg1Δ::natMX atg11Δ::kanMX pha2::P41nmt1-mCherry-atg8(hphMX)(pha2-) leu1-32::Patg1-atg1-Flag-GBP(leu1+) ars1::Patg1-atg1(D193A)-GFP(ura4+)* |
| DY47849 | *h-* | *his3-D1 ura4-D18 atg1Δ::natMX atg11Δ::kanMX pha2::P41nmt1-mCherry-atg8(hphMX)(pha2-) leu1-32::Patg1-atg1(D193A)-Flag-GBP(leu1+) ars1::Patg1-atg1(D193A)-GFP(ura4+)* |
| DY47850 | *h-* | *his3-D1 ura4-D18 atg1Δ::natMX atg11Δ::kanMX pha2::P41nmt1-mCherry-atg8(hphMX)(pha2-) leu1-32::Patg1-atg1(T208A)-Flag-GBP(leu1+) ars1::Patg1-atg1(D193A)-GFP(ura4+)* |
| DY47851 | *h-* | *his3-D1 ura4-D18 atg1Δ::natMX atg11Δ::kanMX pha2::P41nmt1-mCherry-atg8(hphMX)(pha2-) leu1-32::Patg1-atg1-Flag-GBP(leu1+) ars1::Patg1-atg1(T208A)-GFP(ura4+)* |
| DY47852 | *h-* | *his3-D1 ura4-D18 atg1Δ::natMX atg11Δ::kanMX pha2::P41nmt1-mCherry-atg8(hphMX)(pha2-) leu1-32::Patg1-atg1(D193A)-Flag-GBP(leu1+) ars1::Patg1-atg1(T208A)-GFP(ura4+)* |
| DY47853 | *h-* | *his3-D1 ura4-D18 atg1Δ::natMX atg11Δ::kanMX pha2::P41nmt1-mCherry-atg8(hphMX)(pha2-) leu1-32::Patg1-atg1(T208A)-Flag-GBP(leu1+) ars1::Patg1-atg1(T208A)-GFP(ura4+)* |

**Supplementary File 1b. Plasmids used in this study.**

| **Plasmid** | **Descriptive name** | **Description** |
| --- | --- | --- |
| pDB4796 | pDUAL-P41nmt1-mCherry-Atg11 | pDUAL plasmid expressing mCherry-Atg11 from P41nmt1 promoter |
| pDB4797 | pDUAL-P41nmt1-mCherry-Atg11(1-508) | pDUAL plasmid expressing mCherry-Atg11(1-508) from P41nmt1 promoter |
| pDB4798 | pDUAL-P41nmt1-mCherry-Atg11(508-926) | pDUAL plasmid expressing mCherry-Atg11(508-926) from P41nmt1 promoter |
| pDB4799 | pDUAL-P41nmt1-mCherry-Atg11(508-765) | pDUAL plasmid expressing mCherry-Atg11(508-765) from P41nmt1 promoter |
| pDB4800 | pDUAL-Pnmt1-mCherry-Atg11(522-583) | pDUAL plasmid expressing mCherry-Atg11(522-583) from Pnmt1 promoter |
| pDB4801 | pDUAL-Pnmt1-mCherry-Atg11(522-552) | pDUAL plasmid expressing mCherry-Atg11(522-552) from Pnmt1 promoter |
| pDB4802 | pDUAL-Pnmt1-mCherry-Atg11(532-583) | pDUAL plasmid expressing mCherry-Atg11(532-583) from Pnmt1 promoter |
| pDB4803 | pDUAL-Pnmt1-mCherry-Atg11(546-583) | pDUAL plasmid expressing mCherry-Atg11(546-583) from Pnmt1 promoter |
| pDB4804 | pDUAL-P41nmt1-mCherry-Atg11(522-583)Δ | pDUAL plasmid expressing mCherry-Atg11(522-583)Δ from P41nmt1 promoter |
| pDB1626 | pDUAL-P41nmt1-mCherry | pDUAL plasmid expressing mCherry from P41nmt1 promoter |
| pDB4805 | pDUAL-P41nmt1-Pil1-CFP-Atg11(522-583) | pDUAL plasmid expressing Pil1-CFP-Atg11(522-583) from P41nmt1 promoter |
| pDB4806 | pDUAL-P41nmt1-Pil1-CFP-Atg11(522-552) | pDUAL plasmid expressing Pil1-CFP-Atg11(522-552) from P41nmt1 promoter |
| pDB4807 | pDUAL-P41nmt1-Pil1-CFP-Atg11(532-583) | pDUAL plasmid expressing Pil1-CFP-Atg11(532-583) from P41nmt1 promoter |
| pDB4808 | pDUAL-P41nmt1-mCherry-Atg1 | pDUAL plasmid expressing mCherry-Atg1 from P41nmt1 promoter |
| pDB4809 | pDUAL-P41nmt1-mCherry-Atg1(1-311) | pDUAL plasmid expressing mCherry-Atg1(1-311) from P41nmt1 promoter |
| pDB4810 | pDUAL-P41nmt1-mCherry-Atg1(312-592) | pDUAL plasmid expressing mCherry-Atg1(312-592) from P41nmt1 promoter |
| pDB4811 | pDUAL-P41nmt1-mCherry-Atg1(593-830) | pDUAL plasmid expressing mCherry-Atg1(593-830) from P41nmt1 promoter |
| pDB4812 | pGADT7-Atg1 | pGADT7 plasmid expressing GAL4AD-Atg1 from *S. cerevisiae* ADH1 promoter |
| pDB4813 | pGADT7-Atg1(1-592) | pGADT7 plasmid expressing GAL4AD-Atg1(1-592) from *S. cerevisiae* ADH1 promoter |
| pDB4814 | pGADT7-Atg1(593-830) | pGADT7 plasmid expressing GAL4AD-Atg1(593-830) from *S. cerevisiae* ADH1 promoter |
| pLD13 | pGADT7-Crb2(521-778) | pGADT7 plasmid expressing GAL4AD-Crb2(521-778) from *S. cerevisiae* ADH1 promoter |
| pLD5 | pGBKT7-Crb2(276-778) | pGBKT7 plasmid expressing GAL4BD-Crb2(276-778) from *S. cerevisiae* ADH1 promoter |
| pDB4815 | pGBKT7-Atg11(522-583) | pGBKT7 plasmid expressing GAL4BD-Atg11(522-583) from *S. cerevisiae* ADH1 promoter |
| pDB4816 | pGBKT7-Atg11(522-544) | pGBKT7 plasmid expressing GAL4BD-Atg11(522-544) from *S. cerevisiae* ADH1 promoter |
| pDB4817 | pGBKT7-Atg11(546-583) | pGBKT7 plasmid expressing GAL4BD-Atg11(546-583) from *S. cerevisiae* ADH1 promoter |
| pDB4818 | pDUAL-Pnmt1-mCherry-Atg11(522-583) F526A | pDUAL plasmid expressing mCherry-Atg11(522-583) (F526A) from Pnmt1 promoter |
| pDB4819 | pDUAL-Pnmt1-mCherry-Atg11(522-583) Y527A | pDUAL plasmid expressing mCherry-Atg11(522-583) (Y527A) from Pnmt1 promoter |
| pDB4820 | pDUAL-Pnmt1-mCherry-Atg11(522-583) F526AY527A | pDUAL plasmid expressing mCherry-Atg11(522-583) (F526AY527A) from Pnmt1 promoter |
| pDB4821 | pET15b-HA-Atg1(593-830) | pET15b plasmid for expressing His6-HA-Atg1(593-830) from T7/lac promoter in *E. coli* |
| pDB4822 | pETDuet-His6-GST-Atg11(522-544) | pETDuet plasmid for expressing His6-GST-Atg11(522-544) form T7/lac promoter in *E. coli* |
| pDB4823 | pETDuet-His6-GST-Atg11(522-583) F526A | pETDuet plasmid for expressing His6-GST-Atg11(522-544) F526A from T7/lac promoter in *E. coli* |
| pDB4824 | pETDuet-His6-GST-Atg11(522-583) Y527A | pETDuet plasmid for expressing His6-GST-Atg11(522-544) Y527A from T7/lac promoter in *E. coli* |
| pDB4825 | pDUAL-Pnmt1-Atg11(522-583)-GFP | pDUAL plasmid expressing Atg11(522-583)-GFP from Pnmt1 promoter |
| pDB4826 | pDUAL-Pnmt1-mCherry-Atg11(522-552) | pDUAL plasmid expressing mCherry-Atg11(522-552) from Pnmt1 promoter |
| pDB4827 | pDUAL-Pnmt1-Atg11(522-552)-GFP | pDUAL plasmid expressing Atg11(522-552)-GFP from Pnmt1 promoter |
| pDB4828 | pDUAL-Pnmt1-mCherry-Atg11(546-583) | pDUAL plasmid expressing mCherry-Atg11(546-583) from Pnmt1 promoter |
| pDB4829 | pDUAL-Pnmt1-Atg11(546-583)-GFP | pDUAL plasmid expressing Atg11(546-583)-GFP from Pnmt1 promoter |
| pDB4830 | pGADT7-Atg11(522-583) | pGADT7 plasmid expressing GAL4AD-Atg11(522-583) from *S. cerevisiae* ADH1 promoter |
| pDB4831 | pGADT7-Atg11(546-583) | pGADT7 plasmid expressing GAL4AD-Atg11(546-583) from *S. cerevisiae* ADH1 promoter |
| pDB4832 | pGADT7-Atg11(522-552) | pGADT7 plasmid expressing GAL4AD-Atg11(522-552) from *S. cerevisiae* ADH1 promoter |
| pDB4833 | pGBKT7-Atg11(522-552) | pGBKT7 plasmid expressing GAL4BD-Atg11(522-552) from *S. cerevisiae* ADH1 promoter |
| pDB4834 | pETDuet-His6-MBP-Atg11(522-583) | pETDuet plasmid for expressing His6-GST-Atg11(522-583) from T7/lac promoter in *E. coli* |
| pDB4835 | pDUAL-P41nmt1-mCherry-Atg11(522-583) | pDUAL plasmid expressing mCherry-Atg11(522-583) from P41nmt1 promoter |
| pDB4836 | pDUAL-P81nmt1-mCherry-Atg11(522-583)-Leucine_zipper | pDUAL plasmid expressing mCherry-Atg11(522-583)-Leucine_zipper from P81nmt1 promoter |
| pDB4837 | pDUAL-P81nmt1-mCherry-Atg11(522-552)-Leucine_zipper | pDUAL plasmid expressing mCherry-Atg11(522-552)-Leucine_zipper from P81nmt1 promoter |
| pDB4838 | pDUAL-P41nmt1-mCherry-Atg11(532-583)-Leucine_zipper | pDUAL plasmid expressing mCherry-Atg11(532-583)-Leucine_zipper from P41nmt1 promoter |
| pDB4839 | pDUAL-Patg1-mCherry-Atg1 | pDUAL plasmid expressing mCherry-Atg1 from Patg1 promoter |
| pDB4840 | pDUAL-Patg1-mCherry-Atg1-Luecine_zipper | pDUAL plasmid expressing mCherry-Atg1-Leucine_zipper from Patg1 promoter |
| pDB4841 | pPHA2H-P41nmt1-mCherry-Atg8 | pPHA2H plasmid expressing mCherry-Atg8 from P41nmt1 promoter |
| pDB4842 | pPHA2H-P41nmt1-Pho8Δ60(*S. cerevisiae*) | pPHA2H plasmid expressing Pho8Δ60(*S. cerevisiae*) from P41nmt1 promoter |
| pDB4843 | pDUAL-Patg1-GFP | pDUAL plasmid expressing GFP from Patg1 promoter |
| pDB4844 | pDUAL-Patg1-GBP | pDUAL plasmid expressing GBP from Patg1 promoter |
| pDB4845 | pDUAL-Patg1-Atg1-GFP | pDUAL plasmid expressing Atg1-GFP from Patg1 promoter |
| pDB4846 | pDUAL-Patg1-Atg1-GBP | pDUAL plasmid expressing Atg1-GBP from Patg1 promoter |
| pDB4847 | pDUAL-Patg1-Atg1(D193A)-GFP | pDUAL plasmid expressing Atg1(D193A)-GFP from Patg1 promoter |
| pDB4848 | pDUAL-Patg1-Atg1(T208A)-GFP | pDUAL plasmid expressing Atg1(T208A)-GFP from Patg1 promoter |
| pDB4849 | pDUAL-Patg1-Atg1(D193AT208A)-GFP | pDUAL plasmid expressing Atg1(D193AT208A)-GFP from Patg1 promoter |
| pDB4850 | pDUAL-Patg1-Atg1(D193A)-GBP | pDUAL plasmid expressing Atg1(D193A)-GBP from Patg1 promoter |
| pDB4851 | pDUAL-Patg1-Atg1(T208A)-GBP | pDUAL plasmid expressing Atg1(T208A)-GBP from Patg1 promoter |
| pDB4852 | pDUAL-Patg1-Atg1(D193AT208A)-GBP | pDUAL plasmid expressing Atg1(D193AT208A)-GBP from Patg1 promoter |
| pDB4853 | pETDuet-His6-GST-DNLGDSVYMD (peptide S) | pETDuet plasmid for expressing His6-GST-DNLGDSVYMD from T7/lac promoter in *E. coli* |
| pDB4854 | pETDuet-His6-GST-DNLGDAVYMD (peptide A) | pETDuet plasmid for expressing His6-GST-DNLGDAVYMD from T7/lac promoterin *E. coli* |
| pDB4855 | pDUAL-Pnmt1-mCherry-Atg11(546-583)Δ | pDUAL plasmid expressing mCherry-Atg11(546-583)Δ from Pnmt1 promoter |
| pDB4856 | pDUAL-Pnmt1-Atg11(546-583)Δ-GFP | pDUAL plasmid expressing Atg11(546-583)Δ-GFP from Pnmt1 promoter |
| pDB4857 | pDUAL-P41nmt1-mCherry-Atg11(546-583)Δ | pDUAL plasmid expressing mCherry-Atg11(546-583)Δ from P41nmt1 promoter |
